# 1317. *Porphyromonas gingivalis* and *Treponema denticola* Induce Alzheimer's Disease Biomarkers in Mice

**DOI:** 10.1093/ofid/ofad500.1156

**Published:** 2023-11-27

**Authors:** Catherine A Butler, Ali Mohammed, Rita Paolini, Stephanie Gomez, Su Toulson, Eric C Reynolds, Stuart G Dashper

**Affiliations:** University of Melbourne, Melbourne, Victoria, Australia; University of Melbourne, Melbourne, Victoria, Australia; University of Melbourne, Melbourne, Victoria, Australia; University of Melbourne, Melbourne, Victoria, Australia; University of Melbourne, Melbourne, Victoria, Australia; University of Melbourne, Melbourne, Victoria, Australia; University of Melbourne, Melbourne, Victoria, Australia

## Abstract

**Background:**

Periodontitis, a chronic disease that progresses over years, is a modifiable risk factor for Alzheimer’s disease (AD). Periodontitis is caused by endogenous oral pathobionts, including *Porphyromonas gingivalis* (*Pg*) and *Treponema denticola* (*Td*), that have a synergistic relationship resulting in increased virulence. *Pg* and *Td* proteins and DNA have been detected in post-mortem brains of AD patients. However it is unclear if these bacteria infect the brain or if the detected bacterial products are on membrane vesicles (MVs); spherical nanostructures released from bacteria. The aims of this work were to determine whether intact bacterial cells were in brains of mice after chronic oral inoculation with these organisms; whether *Td* enhanced the ability of *Pg* products to penetrate the brain and whether *Td* alone induced AD biomarkers

**Methods:**

C57BL/6 mice were orally inoculated 3 times/week for 12 weeks with either *Pg*, *Td*, or *Pg* + *Td* cells in a 2:1 ratio, or sham inoculated as a negative control. Mice were culled and their brains dissected, with one hemisphere prepared for immunohistochemistry (IHC) as FFPE tissue and probed with antibodies to amyloid beta (Aβ), phospho-Tau (p-Tau) and the *Pg* surface protein RgpA. The other hemisphere had the hippocampus removed for transmission electron microscopy (TEM) analysis.

**Results:**

*Pg* alone and *Pg* + *Td* inoculated mice showed significant increases in alveolar bone loss compared with the uninoculated control. No whole bacterial cells were detected in any of the brains of inoculated mice including those that were IHC positive for RgpA. RgpA immunoactivity was not significantly different in brains of mice that received *Pg* alone or the *Pg*+*Td* combination. Aβ and p-Tau were detected in all mice with an increasing magnitude in uninoculated < *Td* inoculated ≤ *Pg* inoculated ≤ *Pg*+*Td* inoculated.

**Conclusion:**

Conclusions: Repeated oral inoculation of mice with *Pg* alone and *Pg* + *Td* resulted in periodontitis, as determined by alveolar bone loss. The lack of whole bacterial cells in the brain argues against a direct infection mechanism of AD initiation by the oral bacteria *Pg* and *Td*. Our data are consistent with the observed AD-like pathology in the brain resulting from a focal infection of *Pg* and/or *Td* in the mouth mediated by MV penetration of the brain.

**Disclosures:**

**All Authors**: No reported disclosures

